# Formation and Temperature Effect of InN Nanodots by PA-MBE via Droplet Epitaxy Technique

**DOI:** 10.1186/s11671-016-1455-0

**Published:** 2016-05-04

**Authors:** Hugo Juin-Yu Chen, Dian-Long Yang, Tseh-Wet Huang, Ing-Song Yu

**Affiliations:** Department of Materials Science and Engineering, National Dong Hwa University, Hualien, 97401 Taiwan, Republic of China

**Keywords:** Molecular beam epitaxy, Indium nitride, Quantum dots, Reflection high-energy electron diffraction, Droplet epitaxy

## Abstract

In this report, self-organized indium nitride nanodots have been grown on Si (111) by droplet epitaxy method and their density can reach as high as 2.83 × 10^11^ cm^−2^ for the growth at low temperature of 250 °C. Based on the in situ reflection high-energy electron diffraction, the surface condition, indium droplets, and the formation of InN nanodots are identified during the epitaxy. The X-ray photoelectron spectroscopy and photoluminescence measurements have shown the formation of InN nanodots as well. The growth mechanism of InN nanodots could be described via the characterizations of indium droplets and InN nanodots using scanning electron microscopy, atomic force microscopy, and transmission electron microscopy. The density of the InN nanodots was less than that of the In droplets due to the surface diffusion and desorption of atoms during the nitridation and annealing process. The average size and density of InN nanodots can be controlled by the substrate temperatures during the growth. For the growth at lower temperature, we obtained the higher density and smaller average size of InN nanodots. To minimize the total surface energy, the coarsening and some preferred orientations of InN nanodots were observed for the growth at high temperature.

## Introduction

Group III nitride semiconductors attract a great deal of interests for their unique properties such as strong bonding, direct band gap, and good thermal conductivity. Quantum dots (QDs) are quasi-zero-dimensional materials composed of a few molecules [1]. There is a unique characteristic of quantum dots called quantum confinement effect that could make the discontinuous structure easier to be controlled in the wavelength of emitting light. It is a vitally important application in electronic and photoelectric devices for group III nitrides in recent decades. We can adjust the band gap of quantum dots through changing their size. It is also our desire to create a continuous emitting spectrum through the quantum dot technology with various sizes of the quantum dots. It has the potential in quantum dot lasers [2], bioanalysis [3], fluorescence probes [4], quantum dot displays [5], photo-detectors [6], quantum dot memory technologies [7], and so on. Currently, there exist several ways to produce quantum dots such as the chemical colloidal method, lithography, etching, split-gate approach, and the self-assembly method. Conventionally, lithographic technique is the most common way to produce quantum dots. But it is confronted with the challenge that the process of lithographic technique is very complex and time-consuming which does not meet the costs. The self-assembly way to produce quantum dots has gradually been taken seriously for the possibility of being easily fabricated and comparably inexpensive.

For the fabrication of self-assembled indium nitride (InN) quantum dots, several methods have been proposed so far. For example, molecular beam epitaxy (MBE) and metal organic chemical vapor deposition (MOCVD). MBE has the advantage of growth at low temperature [8]. MOCVD is the most common technique in semiconducting materials for high growth rate, easy maintenance, and being suitable for mass production [9]. Although using MBE to grow materials is comparably ineffective and expensive, it nevertheless grows high-quality crystalline thin films. Generally, there are three modes of epitaxy, namely Frank-van der Merwe (FM), Stranski-Krastanov (SK), and Volmer-Weber (VW) modes [10]. O Briot et al. [11] have used MOCVD to grow InN quantum dots. A Yoshikawa et al. [12] and S Gwo et al. [13] fabricated InN quantum dots by MBE via SK mode, the most common way for epitaxy, which demands buffer layers to ensure sufficient lattice match between substrates and epi-layers. It is, however, still difficult to have high quality and simple way to control the growth of self-assembly InN quantum dots. The droplet epitaxy technique was proposed by a Japanese scientist Koguchi in 1990 [14, 15]. In the beginning, droplet epitaxy was applied to produce GaAs crystal [16–18]. Gradually, the range of droplet applications becomes extensive to other semiconductors such as GaN [19–21], InGaN [22], InAs [23], and InN [24, 25]. This state-of-the-art method that gathers attention in recent years is easier than SK mode and is not limited to the lattice parameters. We can therefore choose the substrates freely for droplet epitaxy. The method of indium droplet formation and nitridation to grow InN nanostructures has been employed to fabricate InN quantum dots on various substrates [26–28]. The growth mechanism of InN nanostructures is not as complete as the study of GaAs nanostructures [29]. Currently, most of the studies focused on the nitridation with large flux of In atoms to form InN nanodots on the surface.

The research of droplet epitaxy technique is still far from complete so far. In this letter, we report the InN quantum dots grown on Si (111) substrates with low flux of In droplets and at different substrate temperatures by using plasma-assisted MBE system. Before the formation of In droplets, the surface of Si was served with pre-nitridation treatment to have nitrogen atoms on the surface. Annealing process was also provided after nitridation. In situ reflection high-energy electron diffraction (RHEED) gave us the observation of InN formation process. The characterizations of In droplets and InN nanodots were conducted by field emission scanning electron microscopy (FE-SEM), atomic force microscopy (AFM), X-ray photoelectron spectroscopy (XPS), transmission electron microscopy (TEM), and photoluminescence (PL) in order to study the growth mechanism of InN nanodots by droplet epitaxy. The average size, shape, and density of InN nanodots were investigated at different substrate temperatures.

## Experiment

Droplet epitaxy of InN nanodots was conducted by our ULVAC MBE system with a radio frequency (RF) nitrogen plasma source. Si (111) wafers were cleaned by acetone to remove organic impurity, by 5 % HF solution to remove the native oxide, and then put into the MBE chamber immediately. After the substrate transfer from load lock to growth chamber, the substrate firstly was cleaned thermally at 850 °C for 60 min. Secondly, the pre-nitridation process of Si substrates was kept at 600 °C for 40 min. The nitrogen plasma source was employed under the condition of power 500 W and 6 N N_2_ gas 0.8 sccm which provided beam equivalent pressure (BEP) 1.3 × 10^−5^ Pa. Thirdly, In droplet fomation was conducted at the low flux by Knudsen cell at 775 °C for 30 s (BEP equals to 7.5 × 10^−6^ Pa). Three different substrate temperatures were 250, 350, and 450 °C for the growth of In droplets. Then, the nitridation treatment on the In droplets was 30 min with BEP 1.3 × 10^−5^ Pa to form InN nanodots. Finally, the process of the InN nanodots was in situ annealing at 500 °C for 10 min for recrystallization of InN nanodots and to evaporate the uncrystallized In droplets.

During the droplet epitaxy of InN nanodots, surface condition can be monitored by in situ RHEED with electron beam energy 20 KV in our MBE system. After the growth, the surface morphology of InN nanodots on Si can be observed by a JEOL FE-SEM with accelerating voltage 15 KV which is also used to calculate the average size and density of the InN nanodots in various growth parameters. AFM was employed for the surface roughness and for the observation of the In droplets and InN nanodots. The analysis of surface chemical composition can be conducted by XPS (VGS Thermo K-Alpha). The cross section TEM image of InN nanodots are performed by JEOL JEM-1400 with accelerating voltage 120 KV. PL measurement was conducted at temperature 77 K equipped with a 10-mW and CW 860-nm laser.

## Results and Discussions

### In Situ RHEED Analysis

**Fig. 1:**
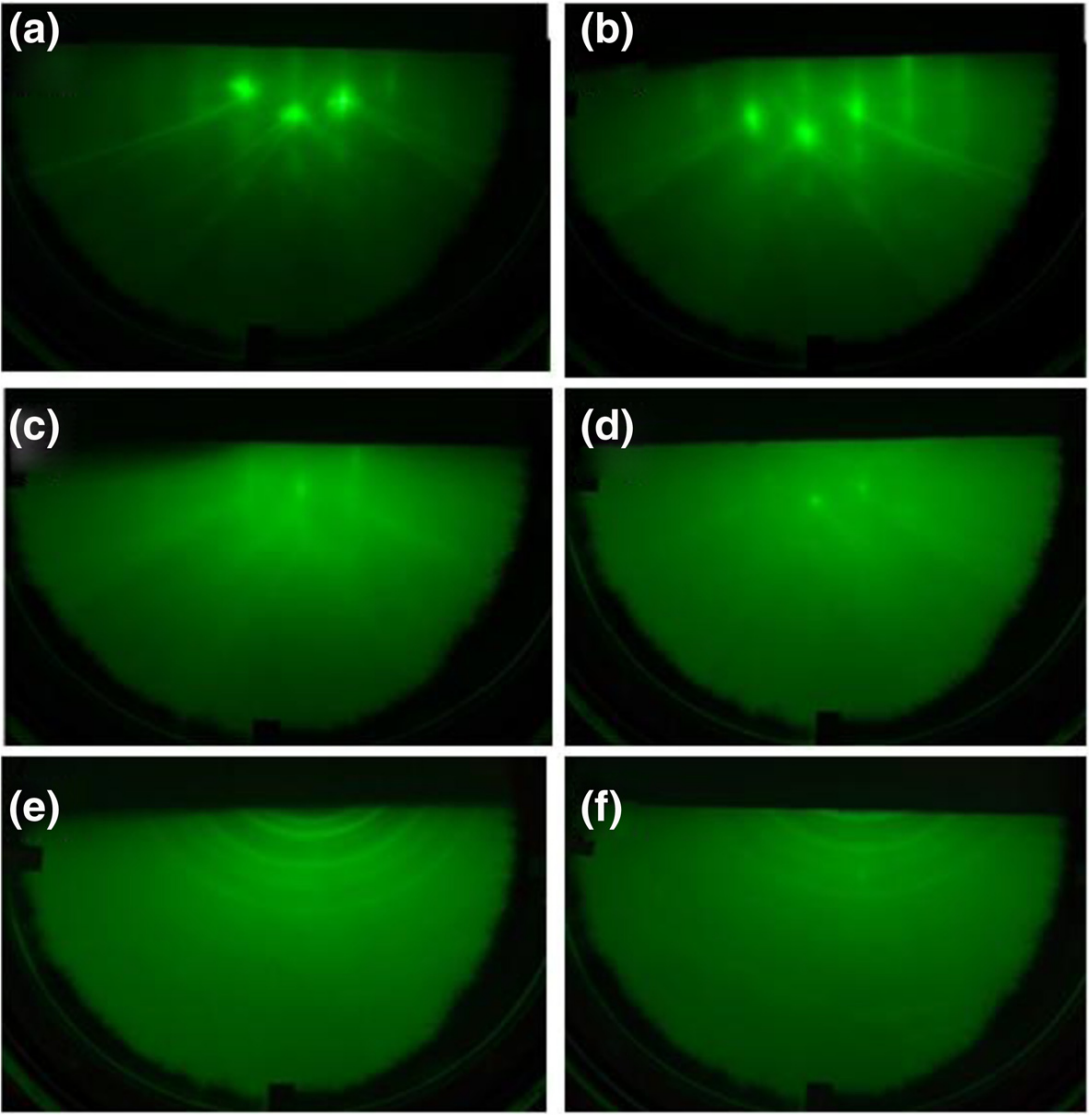
RHEED patterns. **a** Before thermal cleaning process. **b** After thermal cleaning process. **c** After pre-nitridation process. **d** After the formation of In droplets. **e** After the formation of InN nanodots. **f** After annealing process

To identify the condition of sample surfaces and the formation of InN nanodots, high-energy electron beam strikes on the substrate at a tiny angle and the elastically diffracted electrons are monitored by a fluorescent screen, which appears as RHEED patterns as shown in Fig. 1. Before the thermal cleaning, a flat surface gives rise to a long streak in the RHEED pattern as shown in Fig. 1a. After the thermal cleaning, the reconstructed Si surface can be observed and the RHEED pattern is shown in Fig. 1b. After the pre-nitridation treatment, we discover the foggy RHEED pattern in Fig. 1c due to an ultra-thin amorphous SiN_*x*_ layer on the Si wafer. Following the In droplet formation, the RHEED pattern turned spotty and foggy as shown in Fig. 1d, representing the amorphous thin film and rough surface. After the RF plasma nitridation, InN nanodots are formed on the Si substrate. The Debye ring structure of the RHEED pattern indicates random orientation of the InN nanodots on Si (111) as shown in Fig. 1e. After in situ annealing process, the RHEED pattern does not change as shown in Fig. 1f. Based on the results of RHEED, we observe the formation of InN nanodots on the surface of the substrates after the droplet epitaxy.

### The Characterizations of In Droplets and InN Nanodots

**Fig. 2:**
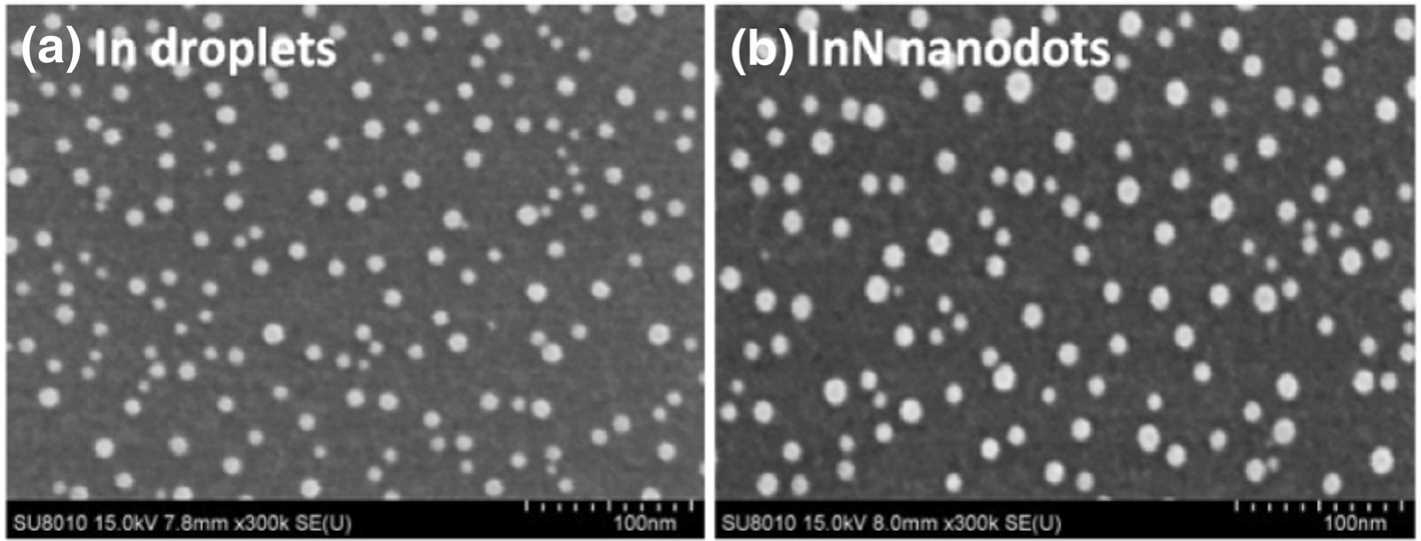
SEM images of In droplets and InN nanodots at substrate temperatures 350 °C. **a** In droplets with the density 1.50 × 10^11^ cm^−2^ and average size 9.4 nm. **b** InN nanodots with the density 1.08 × 10^11^ cm^−2^ and average size 12.3 nm

SEM images of In droplets and InN nanodots are shown in Fig. 2a, b, respectively. After the In droplet formation at the substrate temperature 350 °C, In droplets self-organized on the surface of Si (111), their density is 1.50 × 10^11^ cm^−2^, and the average size is 9.4 nm. After the RF plasma nitridation at the substrate temperature 350 °C, InN nanodots are formed and their density becomes 1.08 × 10^11^ cm^−2^, and average size is 12.3 nm.

**Fig. 3:**
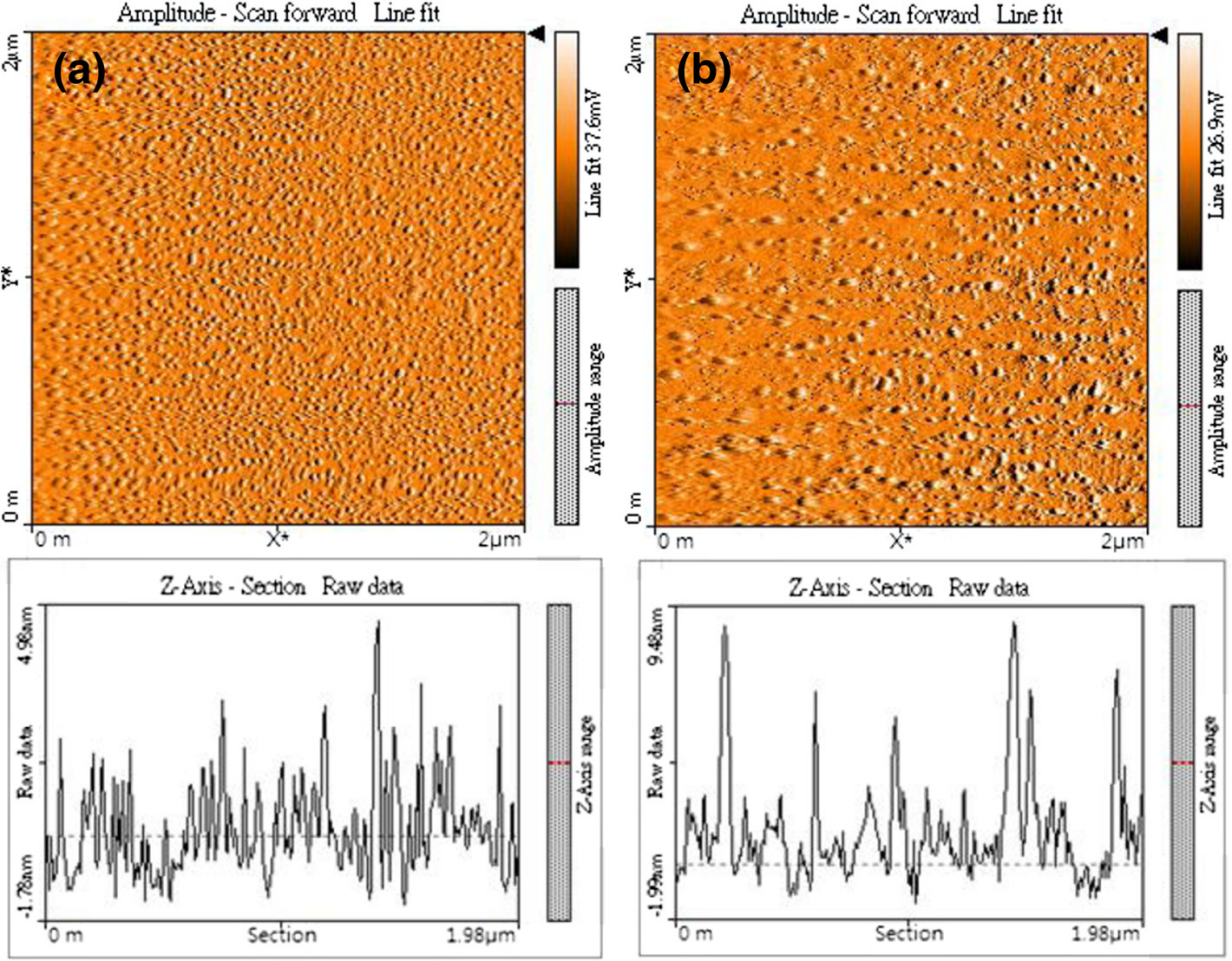
AFM images in amplitudes and line scans of sample surfaces. **a** In droplets with the density 8.64 × 10^10^ cm^−2^. **b** InN nanodots with the density 3.36 × 10^10^ cm^−2^

AFM images in the area 2 μm × 2 μm and 2 μm line scans of sample surface with In droplets and InN nanodots grown at substrate temperature 350 °C are shown in Fig. 3a, b, respectively. We find that the density of In droplets (8.64 × 10^10^ cm^−2^) is higher than that of InN nanodots (3.36 × 10^10^ cm^−2^). Although the density is lower than the results of SEM because of the resolution, the trend is identical to the SEM results. In addition, the root-mean-square values are 1.080 nm for the In droplets and 1.715 nm for the InN nanodots. This implies that the surface roughness and the height of nanodots both increase following the nitridation process.

**Fig. 4:**
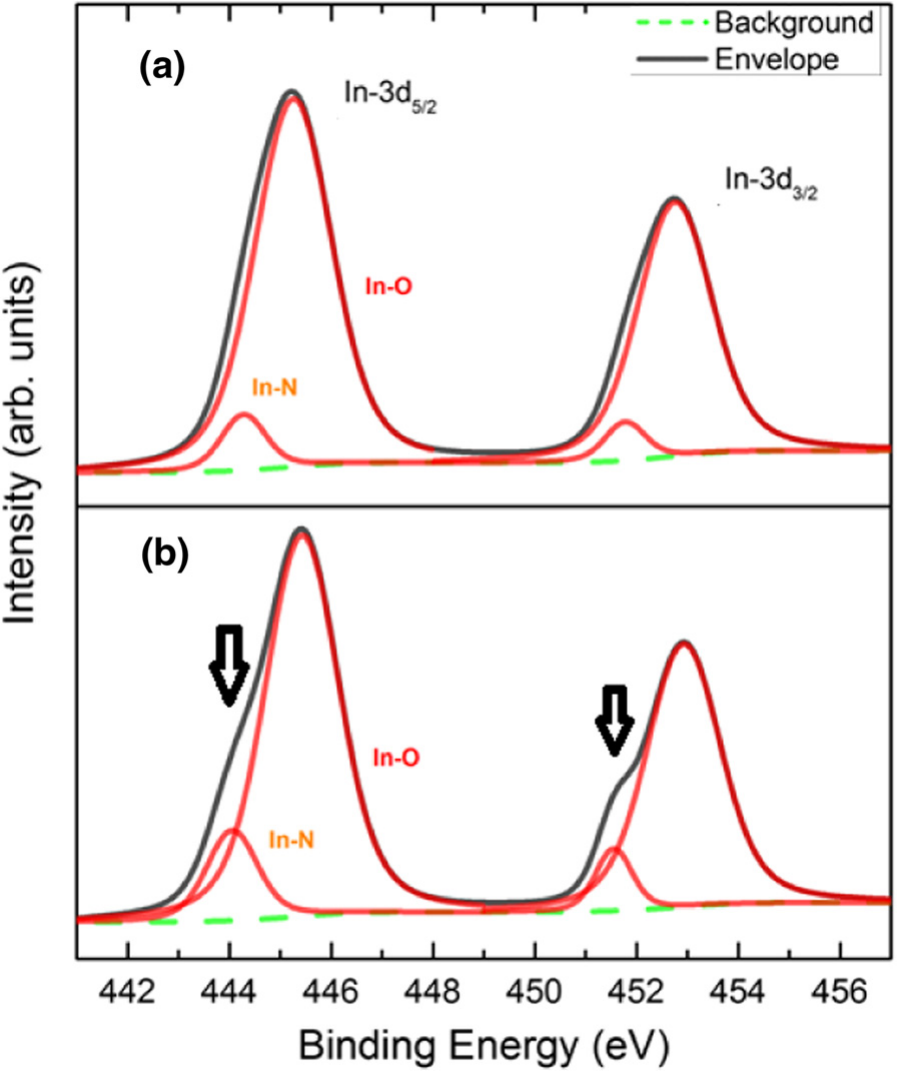
In-3d_5/2_ and In-3d_3/2_ XPS core level spectra. **a** In droplets. **b** InN nanodots

The XPS spectra are carried out in In-3d orbital at the binding energy from 441 to 457 eV, and the software *Avantage* has been used to make the fitting of different bonds. The XPS spectra of the In droplets and InN nanodots are shown in Fig. 4a, b for the samples grown at temperature 350 °C, respectively. In Fig. 4a, the peaks are mainly contributed by the binding energy of In-O due to the oxidation of In droplets in the air. A tiny InN signal comes from the pre-nitridation process of Si substrates. After the formation of InN nanodots, the peaks of In-3d_5/2_ and In-3d_3/2_ become obviously asymmetric due to the binding energy of InN as shown in Fig. 4b. The analysis of surface composition by XPS also shows us the formation of InN nanodots after the droplet epitaxy.

**Fig. 5:**
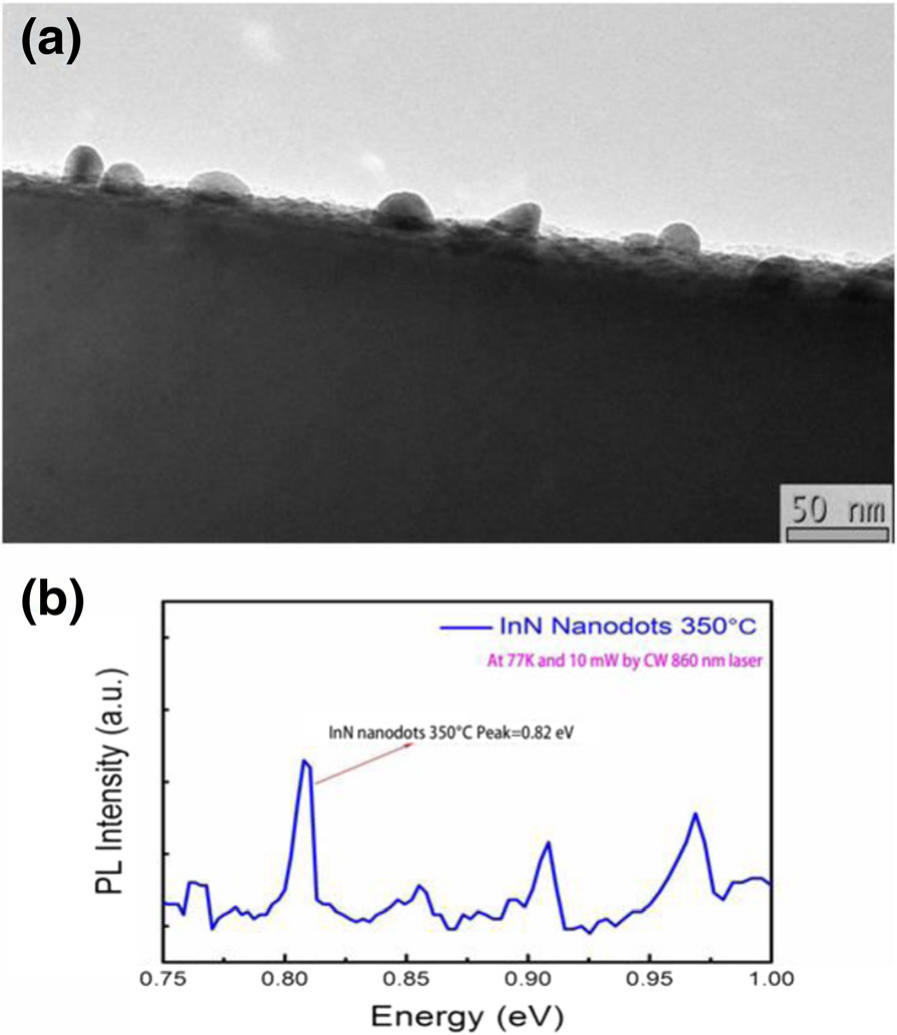
**a** Cross section TEM image of InN nanodots formed at the substrate temperature 350 °C on the Si (111) substrate. The average diameter of InN nanodots is 23.8 nm. **b** PL spectra of InN nanodots with a stronger peak at 0.82 eV

The cross section TEM image of InN nanodots formed at the substrate temperature 350 °C on Si (111) is shown in Fig. 5a. We can clearly find the hemisphere InN nanodots with the average diameter 23.8 nm. Figure 5b shows the PL spectra of InN nanodots with a stronger peak at 0.82 eV.

**Fig. 6:**
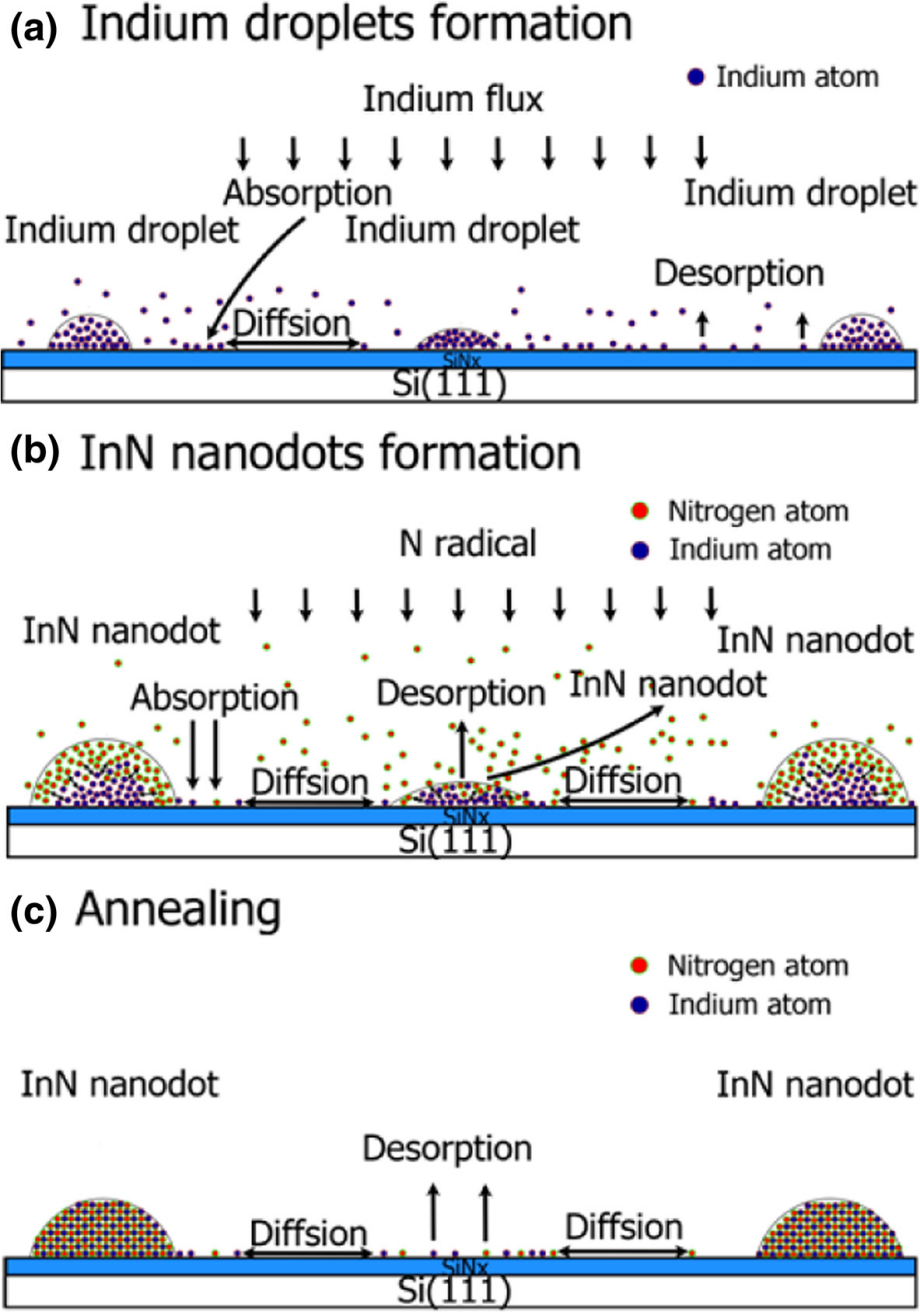
The schematic diagrams of **a** In droplets formation, **b** InN nanodots formation, and **c** annealing process

Based on these analyses, the growth mechanism of InN nanodots on Si (111) by droplet epitaxy could be described by the schematic diagrams in Fig. 6. After thermal cleaning of Si substrates, pre-nitridation treatment provides an ultra-thin amorphous nitride layer on the Si surface. For the formation of the In droplets as shown in Fig. 6a, In droplets are deposited on the surface via the absorption of In atoms, surface diffusion of In atoms, and nucleation of In droplets. Subsequently, during the nitridation process as shown in Fig. 6b, the reaction of indium with nitrogen forms the InN nucleus. During the formation of InN nanodots and annealing process in Fig. 6c, the desorption of indium and nitrogen atoms makes the small In droplets disappear so that lower density and bigger size InN nanodots are observed on the surface after the growth.

### The Effect of Temperatures for InN Nanodots Density

**Fig. 7:**
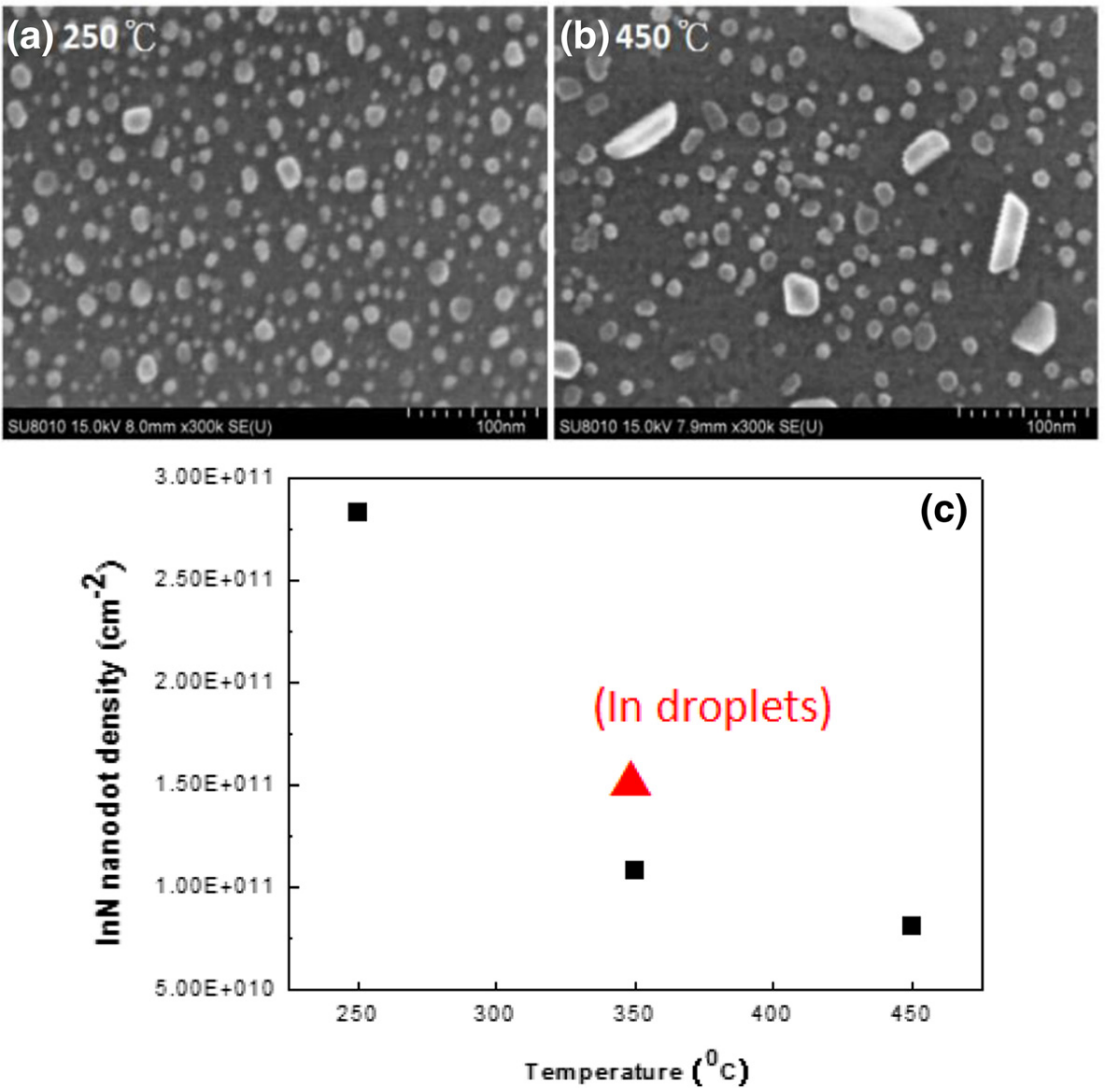
SEM images of InN nanodots at different substrate temperatures. **a** 250 °C, InN nanodots with the density 2.83 × 10^11^ cm^−2^. **b** 450 °C, InN nanodots with the density 8.13 × 10^10^ cm^−2^. **c** Plot of InN nanodot density as a function of substrate temperatures

In this section, we investigate the effect of substrate temperatures with the density of InN nanodots. After thermal cleaning and pre-nitridation, the In droplet formation and nitridation were conducted at substrate temperatures: 250, 350, and 450 °C. According to the SEM images in Fig. 7a, b, we obtained the densities of InN nanodots 2.83 × 10^11^ cm^−2^ and 8.13 × 10^10^ cm^−2^ for the substrate temperatures 250 and 450 °C, respectively. For the growth at 250 °C, InN nanodots have the highest density and smaller average size 10.9 nm. As the substrate temperature increases, the density of InN nanodots becomes lower as shown in Fig. 7c. For the formation of In droplets, the size and density of the In droplets were dependent on the substrate temperature and the flux of In atoms [30]. After nitridation and annealing process, the density could decrease due to desorption of atoms. For the growth at 450 °C, some big InN nanodots were found in irregular shapes in Fig. 7b. To minimize the total surface energy [31], the coarsening and some preferred orientations of InN nanodots were observed after annealing.

**Fig. 8:**
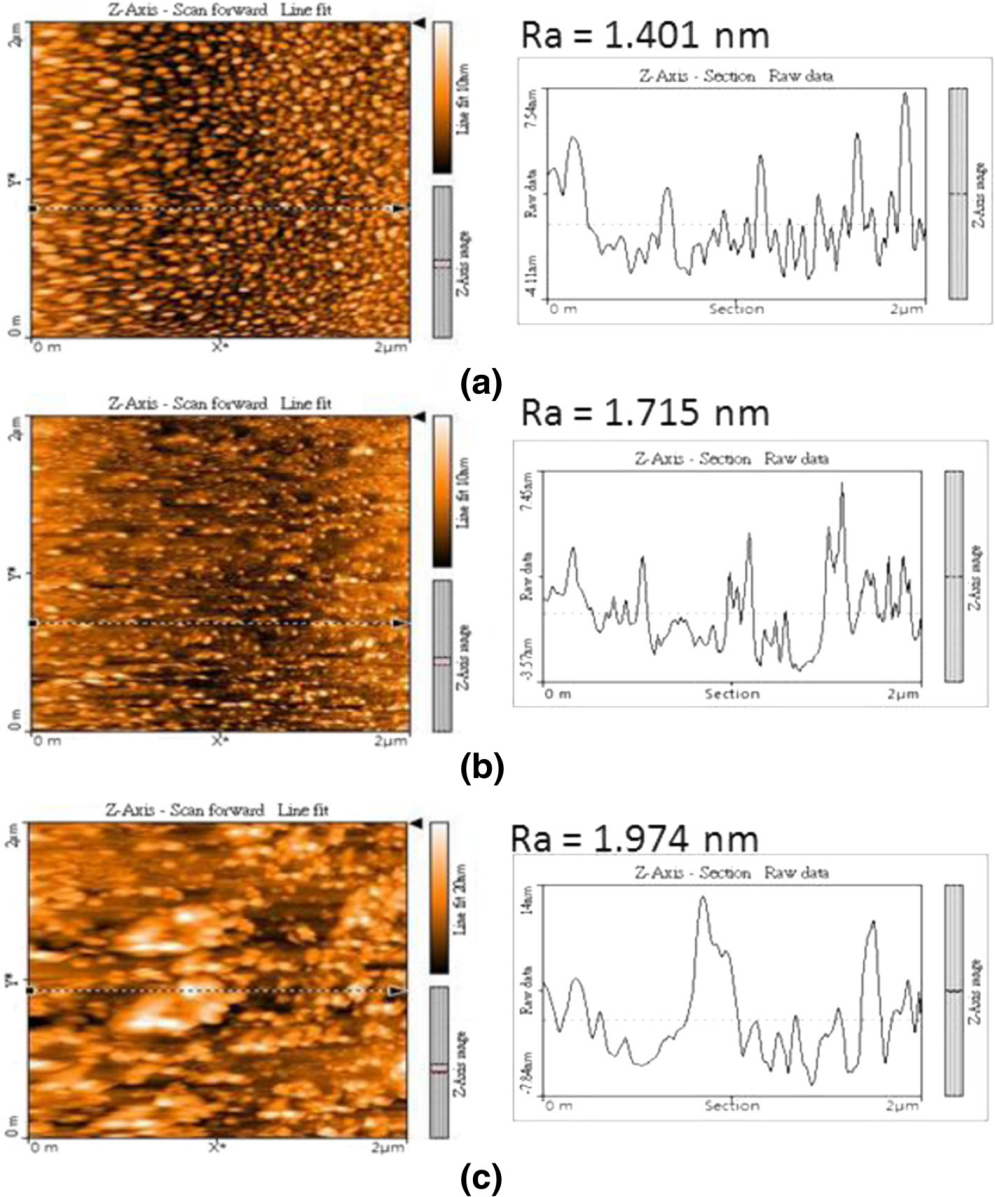
AFM images and line scans of sample surfaces at different growth temperatures. **a** 250 °C. **b** 350 °C. **c** 450 °C

Figure 8a–c performs the AFM images and the line scans for the samples grown at 250, 350, and 450 °C, respectively. The density of InN nanodots decreases while the size of InN nanodots increases as the substrate temperature increases. The root-mean-square values are 1.401 nm for 250 °C, 1.715 nm for 350 °C, and 1.974 nm for 450 °C. The higher substrate temperature, the more apparent surface roughness will be identified. The irregular shapes of InN nanodots were also observed for the growth at 450 °C as shown in Fig. 8c.

**Fig. 9:**
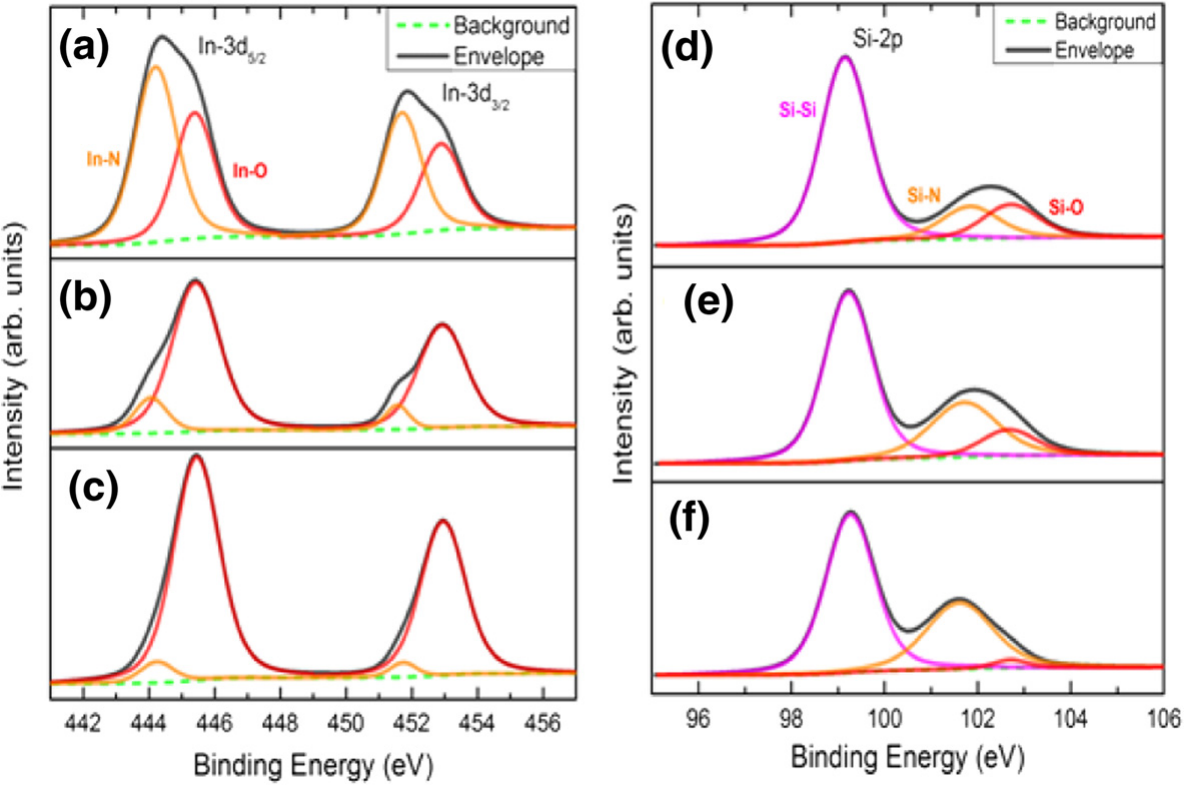
The XPS spectra. **a** In-3d orbital for 250 °C. **b** In-3d orbital for 350 °C. **c** In-3d orbital for 450 °C. **d** Si-2p orbital for 250 °C. **e** Si-2p orbital for 350 °C. **f** Si-2p orbital for 450 °C

To confirm the surface composition of these samples, Fig. 9a–c is the XPS spectra carried out in the binding energy of In-3d orbital. For the substrate temperature 250 °C as shown in Fig. 9a, the XPS spectra perform the strong peaks of InN binding energy. This sample has higher density of InN nanodots, which implies the higher surface coverage by InN nanodots for surface composition analysis. As the temperature increases, the surface coverage by the InN nanodots decreases so that the peaks of InN binding also decrease. Besides, XPS spectra carried out in Si-2p orbital as shown in Fig. 9d–f. The asymmetric peaks are considered the combination of different bonds (Si-Si, Si-N, and Si-O). By separating the characteristic peaks, it is the trend that the higher substrate temperatures, the stronger Si-N signals will be discovered. The reason is attributed to the relatively lower coverage rate of substrate surface by InN nanodots.

## Conclusions

In conclusion, we have fabricated self-organized InN nanodots on Si (111) substrates by PA-MBE via droplet epitaxy technique. The characterizations of InN nanodots and growth mechanism of InN nanodots by droplet epitaxy are discussed in this report according to the analyses of in situ RHEED, SEM, AFM, XPS, TEM, and PL. The formation of InN nanodots is observed by RHEED and XPS. From the results of SEM and AFM, the density of InN nanodots becomes less than that of the In droplets due to the surface diffusion and desorption of atoms during the nitridation and annealing process. The cross section TEM image shows the shape of InN nanodots, and their PL emission performs at the peak energy of 0.82 eV. As the substrate temperatures increase from 250 to 450 °C, the density of InN nanodots decreases while both the average sizes of InN nanodots and surface roughness of the samples increase. For the growth at 250 °C, the density of the InN nanodots can reach as high as 2.83 × 10^11^ cm^−2^. For the growth at 450 °C, the density of the InN nanodots becomes lower and some nanodots are in irregular shapes. The coarsening and some preferred orientations of InN nanodots present to minimize the total surface energy.

## Abbreviations

AFM: atomic force microscopy
FE-SEM: field emission scanning electron microscopy
InN: indium nitride
MBE: molecular beam epitaxy
MOCVD: metal organic chemical vapor deposition
QD: quantum dot
RHEED: reflection high-energy electron diffraction
TEM: transmission electron microscopy
XPS: X-ray photoelectron spectroscopy
